# Ankle Laxity Fluctuation and Sex Hormones in Women with/without Recurrent Sprains

**DOI:** 10.1055/a-2804-6786

**Published:** 2026-02-20

**Authors:** Mako Fukano, Namie Takahashi, Takakuni Sakurai

**Affiliations:** 1 College of Engineering 47745 Shibaura Institute of TechnologySaitamashiSaitamaJapan; 2 Faculty of Health Sciences 146277 Tokyo Ariake Iryo DaigakuKotoTokyoJapan

**Keywords:** ligamentous laxity, relaxin-2, estradiol, menstrual cycle, ankle

## Abstract

The incidence of ankle sprains and chronic ankle instability is higher in women than in men. One sex-specific factor may be the greater ligamentous laxity in women compared with men, potentially influenced by estrogen and relaxin activity. This study aimed to investigate changes in ankle joint laxity and its association with hormone levels in women with and without recurrent ankle sprains. Eleven female college students with recurrent ankle sprains and seven students with no or a single prior sprain participated. Ankle joint laxity was assessed using an ankle arthrometer with a 125-N load for anterior–posterior displacement and with a 4000 N-mm for inversion–eversion rotation. Serum estradiol and relaxin-2 levels were measured. Ankle laxity assessments and blood sampling were performed in the follicular, ovulation, and luteal phases of the participant’s individual menstrual cycle. A significant group×phase interaction was found for inversion–eversion ankle joint laxity; however, post hoc analyses adjusted for Bonferroni multiple comparisons were not significant, implying a lack of clinical interest or potential sampling fluctuations. A positive correlation was found between serum relaxin-2 level and anterior–posterior ankle joint laxity during the luteal phase ( *r* =0.557 and *p* =0.048). This association should be interpreted as an observational finding that may help generate hypotheses regarding potential hormone-related factors in ankle sprains in women.

## Introduction

Ankle sprains are among the most common sport-related injuries and are characterized by ligamentous damage resulting from excessive inversion or eversion, 1​ with a high recurrence rate. 2​ The incidence of ankle sprains and post-traumatic sequelae differs between women and men; in this study, both terms refer to the biological sex assigned at birth. The incidence of ankle sprains is higher in women than in men, 3​ ​4​ and chronic ankle instability, a post-traumatic sequelae, is also more prevalent in women. 5​ One sex-specific risk factor contributing to the higher incidence of ligamentous injuries in women is increased joint laxity, possibly due to hormonal differences and fluctuations across the menstrual cycle.

Fluctuations in estrogen (E2) and progesterone levels across the menstrual cycle have been proposed as a factor contributing to the higher incidence of anterior cruciate ligament (ACL) injuries in women than in men. Previous studies have reported variations in knee laxity throughout the menstrual cycle, with laxity being higher during the ovulatory phase than the menses 6​ and luteal phases. 7​ Certain studies have indicated that ACL injuries are more frequent during the late luteal 8​ or ovulatory phase, 9​ whereas another study reported a higher incidence of injuries on days 1 and 2 of the menstrual cycle. 10​ Additionally, the peptide hormone relaxin-2, which becomes active during the luteal phase, has been the focus of recent research. Relaxin receptors were identified in ACL remnants in female patients but not in male patients undergoing ACL reconstruction, 11​ and female athletes with serum relaxin-2 levels of>6.0 pg/mL had a 4.4-fold increased risk of ACL tears. 12​ These findings suggest that women with elevated relaxin levels during the luteal phase may be at an increased risk for ligamentous injuries. However, it remains unclear whether women with recurrent ankle sprains (RASs) exhibit elevated serum relaxin-2 levels.

Similar to ACL injuries, ankle sprains may be influenced by fluctuations in sex hormones during the menstrual cycle. Evidence regarding the influence of hormonal fluctuations on ankle ligamentous laxity remains limited and inconclusive. Ankle laxity was previously reported to show no significant difference between the pre- and post-ovulatory phases, 13​ and the length of the anterior talofibular ligament did not vary across the menstrual cycle when a 120-N anterior drawer force was applied using specialized equipment. 14​ However, the width of the tibiofibular syndesmosis was found to be greater during the luteal phase than the menstrual, follicular, and ovulation phases. 15​ Furthermore, previous studies did not account for the potential effects of a history of ankle sprain or recurrent injury. To date, no study has examined whether differences in ankle laxity exist between women with and without RASs, and whether these differences are modulated by variations in sex hormone levels. Such information may be valuable for sport professionals involved in conditioning and injury prevention among female athletes, emphasizing the importance of hormonal pre-screening strategies.

The primary aim of this preliminary study was to quantify fluctuations in ankle joint laxity across the menstrual cycle in women with and without a history of RASs. We hypothesized that ankle joint laxity would vary throughout the menstrual cycle and that the patterns of changes would differ between individuals with and without RASs. The secondary aim was to assess potential links between ankle joint laxity and serum levels of E2 and relaxin-2, when available. Furthermore, we hypothesized that elevated sex hormone levels would be associated with increased ankle joint laxity.

## Materials and methods

### Participants

This study was approved by the appropriate ethics committee and conducted in accordance with the principles outlined in the Declaration of Helsinki. This study’s purpose, procedures, and associated risks were explained to all participants, and written informed consent was obtained.

Nineteen collegiate women (individuals assigned female sex at birth) were enrolled; 18 women (mean age, 20.8±1.3 y; height, 156.5±4.4 cm; and weight, 51.4±5.9 kg) completed the study protocol, whereas 1 woman withdrew due to scheduling conflicts. Participants were students enrolled in a health science program and were recruited through the researchers’ personal networks. All participants completed a questionnaire that assessed demographic characteristics, ankle sprain history, menstrual history, pregnancy status, hormonal contraceptive use, and the Japanese version of the Foot and Ankle Ability Measure (FAAM-J). 16​ The FAAM is a self-reported outcome instrument comprising subscales for activities of daily living and sports, used to assess physical functions in individuals with musculoskeletal disorders of the lower leg, foot, or ankles. It has demonstrated reliability, validity, and responsiveness across a broad spectrum of foot and ankle pathologies, 17​ including populations with chronic ankle sprains. 18​ The inclusion criteria included age of 18–25 years and no history of pregnancy or oral contraceptive pill use. The exclusion criteria were any ankle or other injuries within 3 months prior to participation, any neurological or muscular disorders, the presence of subjective symptoms that could interfere with sport activities, or the past or current use of hormonal contraceptives. Participants reported a regular menstrual cycle, with a mean duration of 30±3.9 days. The study design incorporated repeated measures to evaluate within-subject interactions and related factors. A priori sample size calculation with G*Power 3.1 for a mixed (between×within) analysis of variance (ANOVA), assuming Cohen’s *f* =0.4, *α* =0.05, and power=0.80, indicated a minimum requirement of 12 participants. Participants were stratified into two groups based on self-reported ankle sprain history: 11 with two or more previous RASs (RAS group) and 7 with no or only one history of ankle sprain (control group). As the final sample was smaller than the estimated requirement for the intended design, the present study was conducted with limited statistical power.

### Experimental design

All participants attended four laboratory sessions corresponding to the menstrual, follicular, ovulation, and luteal phases of their individual menstrual cycles to complete the experimental protocol. Basal body temperature measurements were used to determine the four menstrual cycle phases. The menstrual phase was defined as the 3 ^rd^ or 4 ^th^ day after menstruation onset, whereas the follicular phase was defined as the 3 ^rd^ or 4 ^th^ day after menstruation cessation. The ovulation phase was defined as the 1 ^st^ or 2 ^nd^ day following the confirmation of a 0.1°C increase in basal body temperatures above baseline. The luteal phase was defined as the period from 5 ^th^ to 10 ^th^ days after the day on which the basal body temperature increased by 0.1°C above baseline. These windows were selected based on the well-described physiological pattern in which serum progesterone level increases shortly after ovulation, leading to a detectable increase in basal body temperature within approximately 24–48 hours. Therefore, the first 1–2 days after the temperature shift correspond to the peri-ovulatory period, whereas days 5–10 reflect the mid-luteal phase when progesterone levels are typically the highest. 19​ Although the basal body temperature does not provide direct information about hormone levels and its charting alone is not a precise indicator of the menstrual cycle phase and may misclassify the timing of ovulation or luteal onset, 20​ the basal body temperature was chosen for its convenience in being easily measured by participants at home and potential utility to identify the proximate day of ovulation.

Blood sampling and ankle laxity assessments were performed during each laboratory visit. To control for diurnal fluctuations in sex hormone levels, blood samples were collected between 10:00 AM and 1:00 PM on each testing day.

### Data collection

Blood samples were collected by a nurse under the supervision of a medical doctor during four laboratory visits aligned with the menstrual cycle and were used to measure serum E2 levels. Serum relaxin-2 levels were obtained from available samples that permitted additional analysis (RAS group: 6 out of 11 samples and control group: 7 out of 7 samples). Samples were left to stand for 20 minutes and then centrifuged for 15 minutes at 4°C. Subsequently, serum components were transferred into specialized containers, frozen at –80°C, and sent to a testing facility (Nikken SEIL CO., Ltd, Japan). Serum E2 levels were measured using an Architect Estradiol II kit (Abbott JAPAN LLC., Japan), and serum relaxin-2 levels were measured using a Relaxin ELISA kit (Immundiagnostik AG., Germany).

Ankle joint laxity was measured using an ankle arthrometer (Blue Bay Research, Inc., Navarre, FL, USA) during each test session. A passive load of 125 N in the anterior and posterior directions and passive inversion–eversion torques of 4,000 N-mm were applied, and the average displacement across the three cycles was calculated. According to a previous study, inter-tester errors were reported to be 1.02 mm for anterior posterior displacement and 2.37 mm for inversion–eversion rotation, which were within the acceptable range of laxity values; 21​ therefore, to eliminate inter-rater variability, all ankle laxity assessments were performed by a single examiner using a previously validated protocol. 22​ The assessment of ankle ligamentous laxity using this instrument has been demonstrated to be both reliable and valid. 21​ ​22​ Data from the dominant ankle of each participant were analyzed.

Regarding blinding, investigators who measured serum concentrations were blinded to both the menstrual cycle phase of the participants and their group allocation, and researchers who assessed ankle laxity were blinded to the participants’ group assignment.

### Statistical analyses

Data were summarized as means and standard deviations using SPSS version 23 (IBM Corp., Armonk, NY, USA). The normality and homogeneity of each variable were assessed using the Shapiro–Wilk test. An independent two-tailed *t* -test was used to compare demographic characteristics between the RAS and control groups. A mixed-design ANOVA (group×phase) was conducted to evaluate ankle laxity and E2 levels during the menstrual cycle. Bonferroni’s post-hoc analysis was performed if the ANOVA showed significant main and interaction effects. Pearson’s product–moment correlation coefficients were calculated to examine the associations between ankle laxity and serum hormone levels. Spearman’s rank correlation coefficient was used for non-normally distributed data, as determined by the Shapiro–Wilk test. Statistical significance was defined as *p* <0.05.

## Results

The demographic characteristics of the participants are presented in Table 1 .

**Table:** **Table 1** Demographic characteristics of the participants

Group	RAS group ( *n* =11)		Control group ( *n* =7)		
Variable	Mean	(SD)	Mean	(SD)	*p* value
Ankle sprain episodes (times)	5	5.19	0.57	0.53	0.041*
Age (years)	20.9	1.58	20.1	0.69	0.246
Height (cm)	156.3	4.84	157.0	4.64	0.761
Weight (kg)	51.2	6.23	53.3	10.88	0.604
FAAM-ADL	98.9	1.4	100	0	0.024*
FAAM-sports	96.3	6.7	100	0	0.097

Abbreviations: ADL, activities of daily living; FAAM, foot and ankle ability measure; RAS, recurrent ankle sprain; SD, standard deviation.

*Significant difference.

The two-way ANOVA indicated no significant main effects of group ( *F* =1.170, *p* =0.331, and partial *η* ²=0.068) or phase ( *F* =0.446 and *p* =0.514), and no interaction effect ( *F* =2.129, *p* =0.109, and partial *η* ²=0.117) for anterior–posterior ankle joint laxity ( Fig. 1 ). For inversion–eversion ankle joint laxity, the main effects of group ( *F* =0.409, *p* =0.531, and partial *η* ²=0.025) and phase ( *F* =1.109, *p* =0.355, and partial *η* ²=0.065) were not significant; however, the interaction effect was significant ( *F* =3.984, *p* =0.013, and partial *η* ²=0.199). Given the potential influence of the group×phase interaction, simple main effects were prioritized and analyzed. A significant simple main effect of the phase was observed within the RAS group ( *F* =2.99, significance of *F* =0.040, and partial *η* ²=0.124); therefore, Bonferroni-adjusted post hoc comparisons did not reach statistical significance (significance of *F* > *α* , *α* =0.0083; Fig. 1 ).

**Fig. 1:**
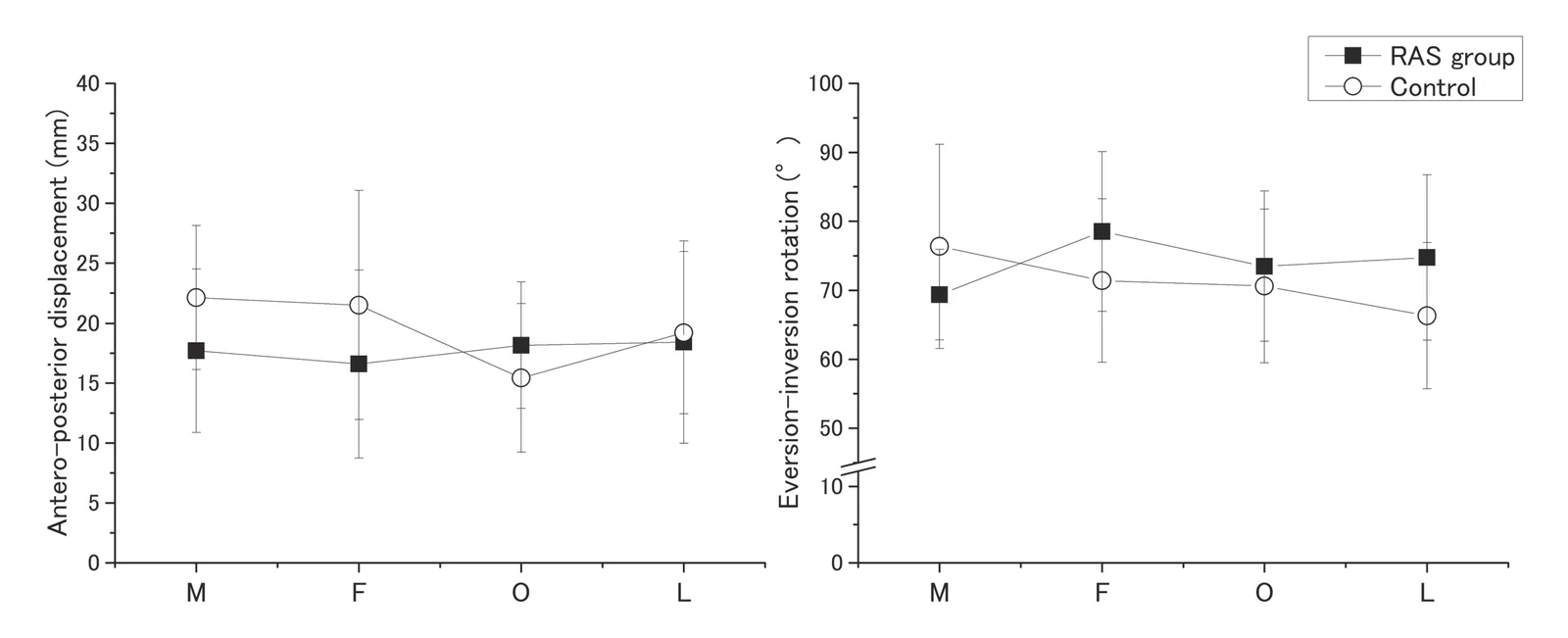
Changes in ankle joint laxity across the menstrual cycle, presented as anterior–posterior displacement (left) and eversion–inversion rotation (right). Data are expressed as means±standard deviations. F, follicular phase; L, luteal phase; M, menstrual phase; O, ovulatory phase; RAS, recurrent ankle sprain.

No significant intergroup differences in E2 levels ( *F* =0.117, *p* =0.737, and partial *η* ²=0.107) or interaction effects ( *F* =0.046, *p* =0.833, and partial *η* ²=0.003) were observed; however, a significant main effect of the phase was present ( *F* =9.333, *p* <0.001, and partial *η* ²=0.368). Bonferroni’s post-hoc analysis was conducted due to the presence of a significant main effect and absence of interaction effects in the ANOVA. E2 levels were significantly higher during the ovulation phase ( *p* =0.035) and luteal phase ( *p* =0.008) than the menstrual phase, and higher in the luteal phase versus the follicular phase ( *p* =0.0012; Fig. 2 ).

**Fig. 2:**
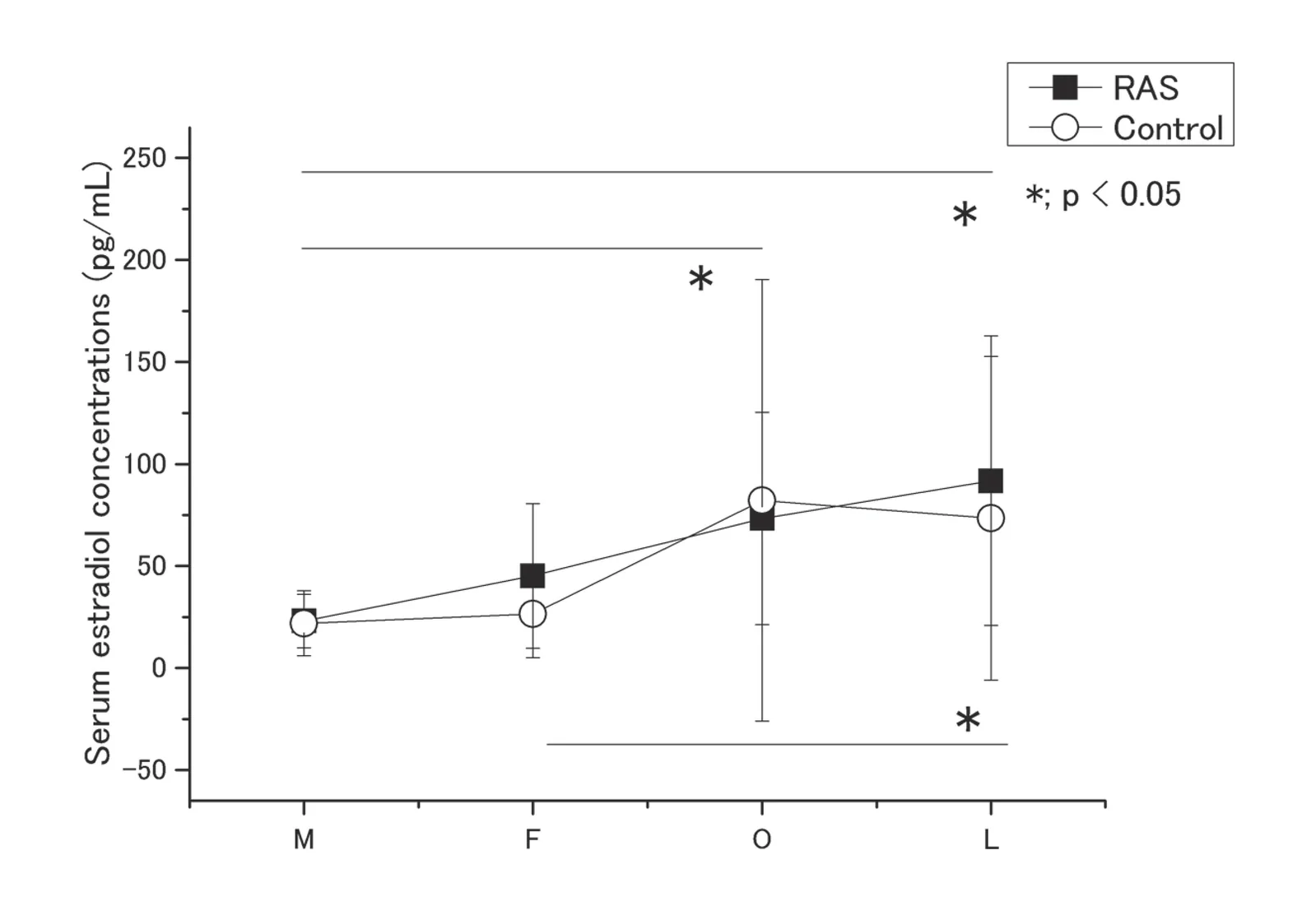
Changes in the serum estradiol (E2) levels across the menstrual cycle. Data are expressed as means±standard deviations. F, follicular phase; L, luteal phase; M, menstrual phase; O, ovulatory phase; RAS, recurrent ankle sprain.

Table 2 presents the Pearson or Spearman correlation coefficients between serum E2 (pg/mL) and serum relaxin-2 (pg/mL) levels and ankle joint laxity across menstrual cycle phases. Significant correlations were found between serum relaxin-2 levels in the follicular phase and anterior–posterior ankle joint laxity during the ovulatory phase, as well as between the serum relaxin-2 level and anterior–posterior ankle joint laxity within the luteal phase.

**Table:** **Table 2** Correlations between serum levels of estradiol and relaxin-2 and ankle joint laxity

	Phase	Ankle laxity (anterior–posterior displacement)								Ankle laxity (eversion-inversion)							
		*M*		*F*		*O*		*L*		*M*		*F*		*O*		*L*	
Variable		Pearson correlation	Sig	Pearson correlation	Sig	Pearson correlation	Sig	Pearson correlation	Sig	Pearson correlation	Sig	Pearson correlation	Sig	Spearman correlation	Sig	Pearson correlation	Sig
Estradiol	M	0.084	0.740	−0.261	0.296	−0.392	0.107	0.053	0.836	0.152	0.547	−0.039	0.879	0.295	0.328	0.140	0.579
	F	0.162	0.521	−0.005	0.985	0.044	0.863	0.202	0.422	−0.013	0.958	0.190	0.449	−0.102	0.741	0.248	0.321
*n* =18	O	0.051	0.839	−0.110	0.663	−0.128	0.614	0.228	0.364	−0.288	0.246	−0.087	0.731	0.429	0.144	−0.269	0.281
	L	−0.081	0.749	−0.288	0.246	−0.291	0.241	0.230	0.360	−0.016	0.949	0.185	0.463	0.003	0.993	0.120	0.635
Relaxin 2	M	0.427	0.145	0.125	0.685	−0.245	0.421	0.350	0.241	0.381	0.199	−0.296	0.326	−0.316	0.201	−0.169	0.582
	F	−0.095	0.756	−0.294	0.329	−0.587	**0.035** *	0.026	0.933	0.438	0.134	−0.208	0.496	−0.012	0.961	0.076	0.804
*n* =13	O	−0.018	0.953	−0.118	0.701	0.125	0.684	−0.420	0.153	0.318	0.290	0.391	0.187	−0.451	0.060	0.072	0.814
	L	0.213	0.485	0.285	0.345	0.179	0.558	**0.557**	**0.048** *	0.141	0.646	0.069	0.822	−0.324	0.189	0.089	0.773

Abbreviations: F, follicular phase; L, luteal phase; M, menstrual phase; O, ovulatory phase; sig, significance.

* *p* <0.05​​​​​.

## Discussion

The present study found no fluctuation in ankle joint laxity across the menstrual cycle in women with and without RASs, although a potential association between sex hormones and the degree of ankle joint laxity was indicated.

Ankle joint laxity in response to applied stress did not fluctuate across the menstrual cycle, which is consistent with previous studies on women with no history of lower extremity injury 13​ ​23​ and with no history of ankle sprains in the past 6 months. 14​ A novel finding of this study is that ankle laxity remains stable throughout the menstrual cycle in individuals with or without a history of RASs, as no change was observed in either the RAS or control group. Although a significant group×phase interaction was detected, Bonferroni-adjusted post hoc analysis were not significant, suggesting limited clinical relevance and possible sampling-related fluctuations. However, the FAAM scores indicated relatively mild symptoms, suggesting that future studies should consider the severity of prior injuries. Additionally, a previous study reported increased tibiofibular syndesmosis width during the luteal phase. 15​ Further studies are warranted to evaluate ligaments, specifically to determine the presence of estrogen and/or relaxin-2 receptors.

Significant correlations were found between serum relaxin-2 levels in the follicular phase and anterior–posterior ankle joint laxity in the ovulatory phase, as well as between serum relaxin-2 levels in the luteal phase and anterior–posterior ankle joint laxity in the luteal phase ( Fig. 3 ). The serum relaxin-2 level in the follicular phase and anterior–posterior ankle joint laxity in the ovulatory phase were negatively correlated, indicating that participants with higher relaxin-2 levels in the follicular phase exhibited reduced ankle joint laxity in the ovulatory phase. This finding contradicts the known actions of relaxin, which may stimulate collagenase expression, suppress collagen synthesis, 24​ and decrease mechanical properties. 25​ In contrast, the serum relaxin-2 level and anterior–posterior ankle joint laxity in the luteal phase showed a positive correlation, potentially influenced by outliers with elevated relaxin-2 levels. Although the participant had no reported history of ankle sprain at this time, her serum relaxin level exceeded 6.0 pg/mL, a level that has been associated with a higher reported risk of ACL injury. 12​ Therefore, our observation may provide a basis for generating hypotheses regarding hormone-related contributors to ligamentous injury. Importantly, as this study did not track subsequent ankle sprain incidence, our interpretation represents an extrapolation from the ACL literature rather than evidence of an established mechanism for ankle sprains. Nevertheless, this preliminary study suggests a possible association between the serum relaxin level and ankle laxity in the luteal phase, warranting further investigation with a larger cohort. Epidemiological studies are also required to determine the association between ankle sprain incidence and different menstrual cycle phases.

**Fig. 3:**
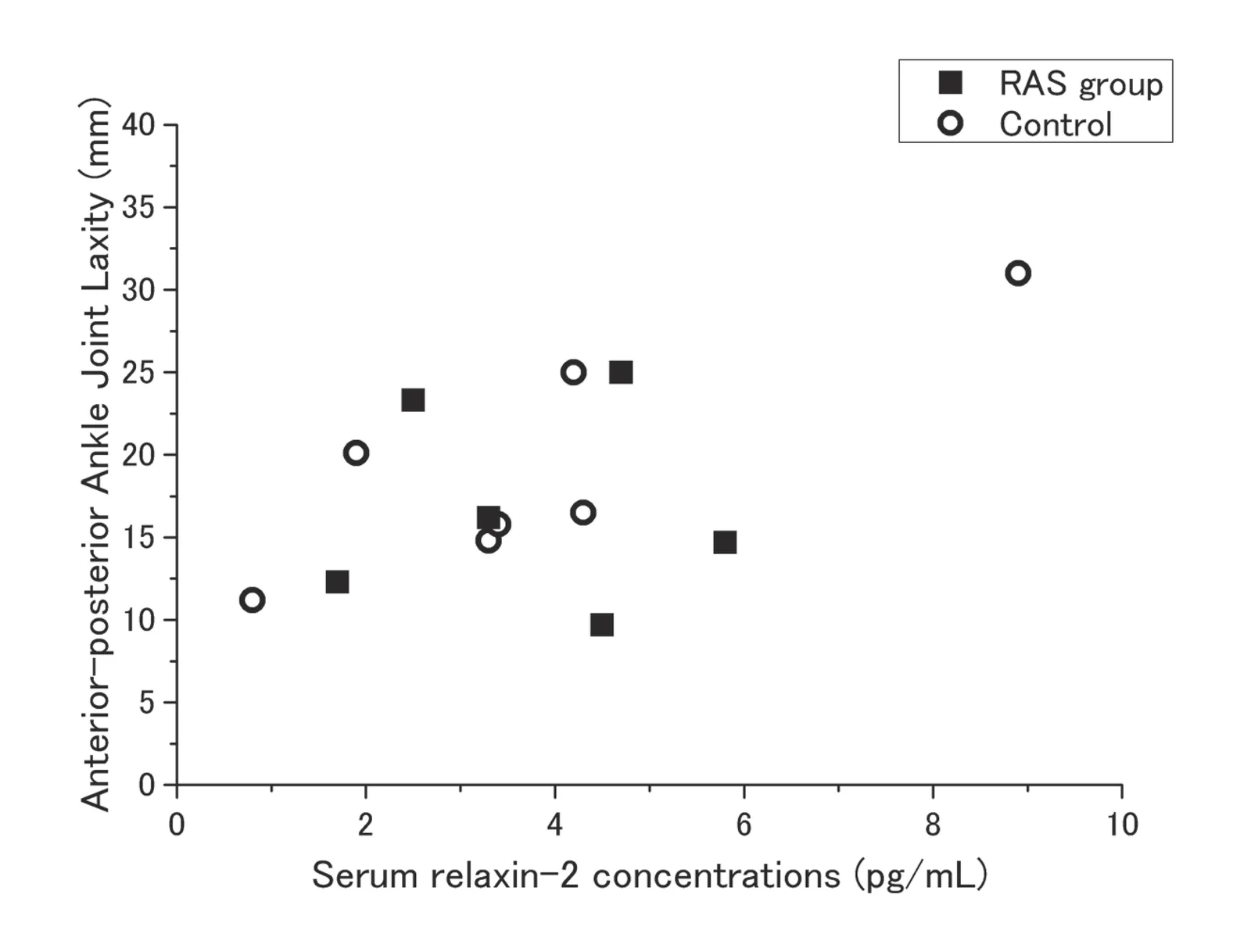
Association between the serum relaxin-2 level and ankle anterior–posterior displacement during the luteal phase. RAS, recurrent ankle sprain.

This preliminary study investigated fluctuations in ankle laxity across the menstrual cycle in women with and without RASs and examined the associations between serum levels of E2 and relaxin-2 and ankle laxity. This study has some limitations. First, the sample size was relatively small due to its preliminary nature. This may have reduced the ability to detect interaction effects in the two-way ANOVA and, at the same time, may have resulted in unstable estimates that should be interpreted with caution. In addition, because the final sample size did not reach the predetermined target, the present findings should be regarded as hypothesis-generating rather than confirmatory. Second, there are methodological limitations. Serum sex hormones were measured using ELISA, which is less accurate than mass spectrometry, the gold standard. The basal body temperature is also a limited tool for determining the menstrual cycle phase. In addition, grouping was based on self-reported injury history, which may be subjected to recall bias. Third, all participants were college-aged women, limiting the generalizability of the findings to other age groups.

## Conclusions

The present study demonstrated variations in ankle laxity across the menstrual cycle and their associations with serum levels of E2 and relaxin-2. A significant group×phase interaction was found for inversion–eversion ankle joint laxity; however, post hoc analyses adjusted for Bonferroni multiple comparisons were not significant, implying a lack of clinical interest or potential sampling fluctuations. In the luteal phase, we observed potential associations between increased anterior–posterior ankle joint laxity and elevated relaxin-2 levels. These findings provide preliminary observational insights that could inform hypotheses about potential hormone-related influences on ankle sprains in women.
